# Update on Lynch syndrome genomics

**DOI:** 10.1007/s10689-016-9882-8

**Published:** 2016-02-12

**Authors:** Päivi Peltomäki

**Affiliations:** Department of Medical and Clinical Genetics, University of Helsinki, P. O. Box 63, Haartmaninkatu 8, 00014 Helsinki, Finland

**Keywords:** Lynch syndrome, Mutation, Epimutation, Tumor spectrum, DNA mismatch repair

## Abstract

Four main DNA mismatch repair (MMR) genes have been identified, *MLH1*, *MSH2*, *MSH6*, and *PMS2*, which when mutated cause susceptibility to Lynch syndrome (LS). LS is one of the most prevalent hereditary cancer syndromes in man and accounts for 1–3 % of unselected colorectal carcinomas and some 15 % of those with microsatellite instability and/or absent MMR protein. The International Society for Gastrointestinal Hereditary Tumours (InSiGHT) maintains a database for LS-associated mutations since 1996. The database was recently reorganized to efficiently gather published and unpublished data and to classify the variants according to a five-tiered scheme linked to clinical recommendations. This review provides an update of germline mutations causing susceptibility to LS based on information available in the InSiGHT database and the latest literature. MMR gene mutation profiles, correlations between genotype and phenotype, and possible mechanisms leading to the characteristic spectrum of tumors in LS are discussed in light of the different functions of MMR proteins, many of which directly serve cancer avoidance.

## DNA mismatch repair genes: shared and specialized functions

Functional DNA mismatch repair (MMR) is vital for basic biology and cancer avoidance. The main function of MMR proteins is to maintain genomic stability by correcting single-base mismatches and insertion/deletion loops (IDL) that may arise during replication [1]. Malfunction of MMR results in a mutator phenotype and microsatellite instability (MSI) characteristic of most tumors from Lynch syndrome (LS) and some 15 % of sporadic tumors [2]. MMR proteins also recognize diverse types of endogenous and exogenous damage, such as that induced by oxidation [3] or alkylation [4], and correct the lesions, or if this is not possible, signal DNA damage to cell cycle arrest or apoptosis. MMR proteins regulate genetic recombination by correcting mismatches that may occur in recombination during meiosis and by suppressing recombination between homeologous (=related but non-identical) sequences during mitosis [5]. Unexpectedly, the MMR system can also promote mutations when needed. For example, the MMR proteins MSH4 and MSH5 facilitate meiotic crossover between homologous chromosomes [6]. Additionally, MMR proteins promote somatic hypermutation and class switch of antibody genes [7].

**Fig. 1:**
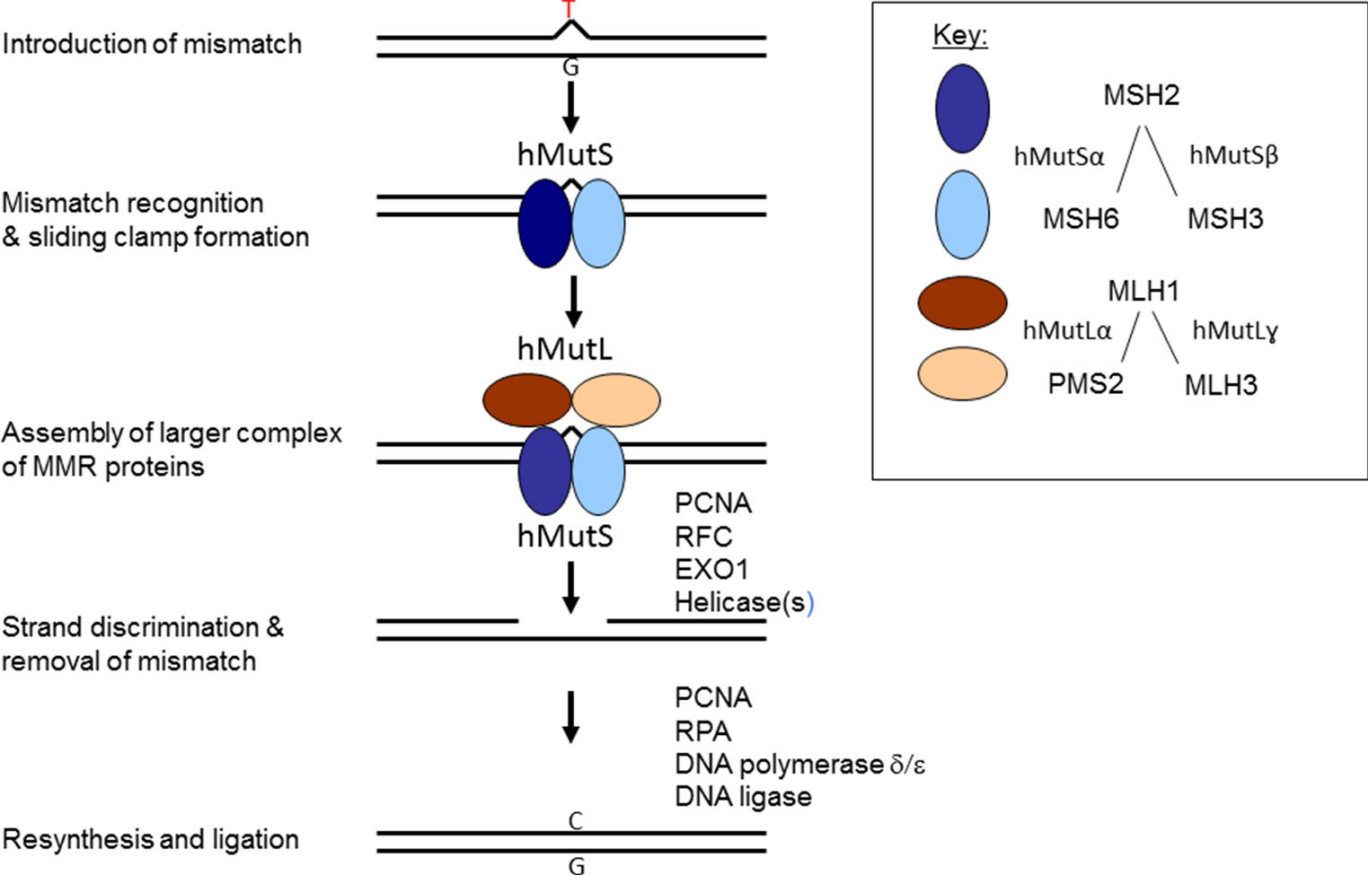
The different hMutS and hMutL complexes in human MMR. In addition to MMR proteins, the repair process requires a number of other proteins, such as proliferating cell nuclear antigen (PCNA), replication factor C (RFC), EXO1 (a 5′–3′ exonuclease), DNA helicases, RPA (replication protein A, a single-stranded DNA binding protein), DNA polymerases, and DNA ligase

In humans, five MutS homologues (MSH2, MSH6, MSH3, MSH4, and MSH5) and four MutL homologues (MLH1, PMS2, PMS1, and MLH3) have been identified which can form heterodimers in different combinations [8–10] (Fig. 1). The main mismatch-binding factor in humans is hMutSα, consisting of MSH2 and MSH6, which recognizes single-base mispairs and IDLs. Another mismatch-binding heterodimer is hMutSβ, formed by MSH2 and MSH3, which mainly acts on IDLs. Upon mismatch binding, the hMutS complex undergoes an ATP-driven conformational change into a sliding clamp and a hMutL heterodimer is recruited. The main hMutL complex is hMutLα, consisting of MLH1 and PMS2 and participating in the repair of single-base mismatches and IDLs. Alternative hMutL heterodimers are hMutLγ, composed of MLH1 and MLH3, which may predominantly contribute to IDL repair, and hMutLβ (MLH1 and PMS1), which does not seem to participate in MMR. When the hMutS-hMutL complex encounters a strand discontinuity, an excision machinery is recruited, the mismatch containing fragment is degraded, and a new strand synthesized [7, 9].

Substrate specificities of the individual MMR proteins are reflected in the different MSI phenotypes observed in tumors from LS patients. *MSH2* and *MLH1* mutations are associated with high-degree instability involving mononucleotide and dinucleotide (and other short tandem) repeats [11]. The same is true for *PMS2* mutations [12]. *MSH6* mutations are associated with low-degree MSI with a preferential involvement of mononucleotide repeats [13]. In tumors from *MLH3* mutation carriers, mononucleotide repeats may be less informative than dinucleotide and tetranucleotide repeats [14] and phenotypes ranging from MSI-high [14] to no MSI [15] have been reported.

## Germline mutations in MMR genes predisposing to LS

### Shares of individual MMR genes

*MLH1*, *MSH2*, *MSH6* and *PMS2* account for 40, 34, 18, and 8 %, respectively, of the 3000 unique germline sequence variants of MMR genes deposited to the International Society for Gastrointestinal Hereditary Tumours (InSiGHT) database ([16] and www.insight-group.org, date accessed December 19th, 2015). The different substrate specificities described above may explain why *MLH1* and *MSH2* are the most important predisposing genes for LS (their protein products are obligatory components in all types of heterodimers, Fig. 1), followed by *MSH6* and *PMS2*, whereas *MLH3* mutations are rare (functionally redundant with *PMS2*), and no LS-predisposing germline mutations are known for *MSH3* (functionally redundant with *MSH6*). No LS-associated germline mutations have been detected in *MSH4* or *MSH5* (their primary role is in meiotic recombination rather than MMR).

### Mutation spectra

**Fig. 2:**
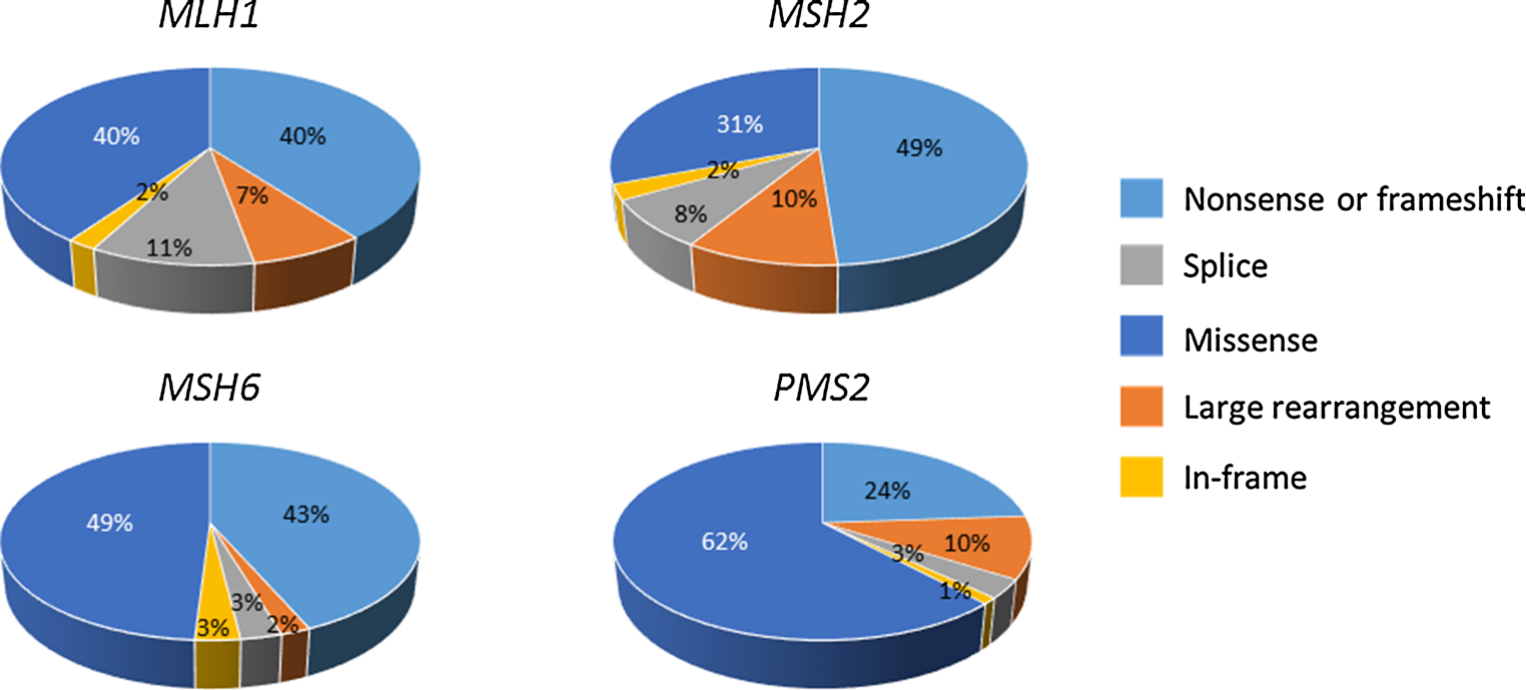
Distributions of the types of germline variants across each MMR gene. The analysis is based on data deposited in the InSiGHT database [17] and is restricted to variants with coding changes. The total numbers of variants per gene included in the analysis are 1104 for *MLH1*, 883 for *MSH2*, 414 for *MSH6*, and 197 for *PMS2*

**Fig. 3:**
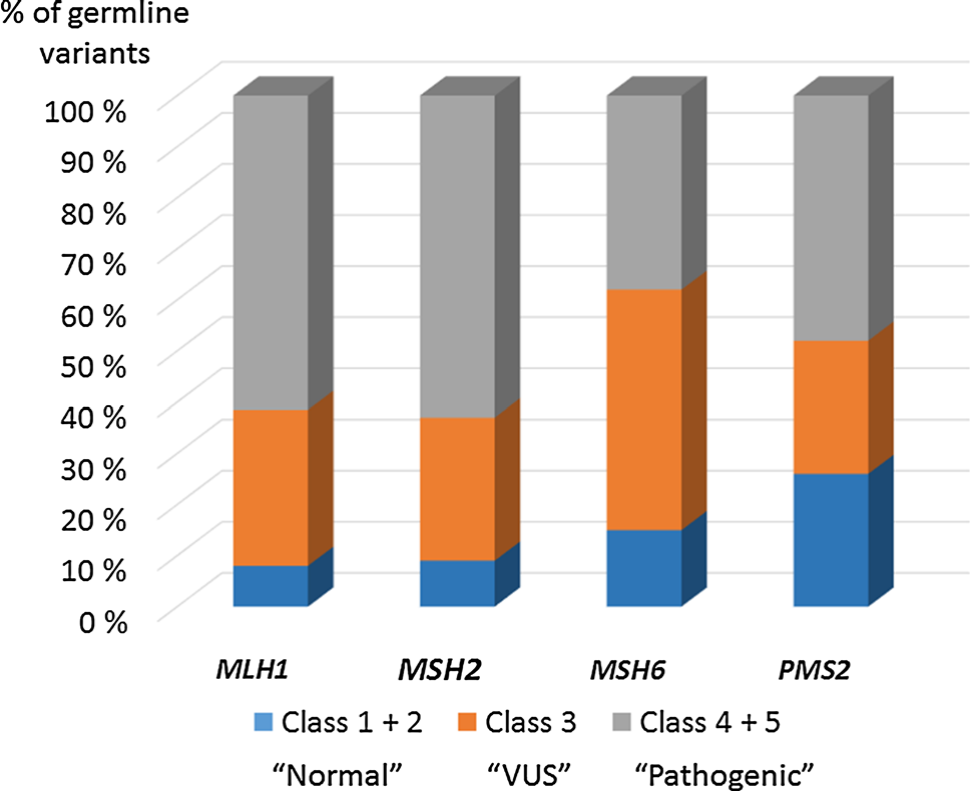
Distributions of the different pathogenicity classes within the LS-associated MMR genes. The relative shares of normal variants (pathogenicity classes 1 and 2), VUSes (class 3), and pathogenic mutations (classes 4 and 5) reported for each MMR gene in the InSiGHT database [16] are depicted. The analysis includes 932 sequence variants for *MLH1*, 842 for *MSH2*, 449 for *MSH6*, and 137 for *PMS2*

Mutations are scattered throughout the MMR genes (www.insight-group.org). Figure 2 displays the gene-specific distributions of germline variants by the type of mutation and predicted coding change [17]. Most *MLH1*, *MSH2*, and *MSH6* mutations are truncating (predominantly nonsense or frameshift mutations). Moreover, the share of missense changes, which lead to single amino acid substitutions, is significant (~30–60 %) for all four genes. The abundance of missense mutations prompted the InSiGHT to undertake a large-scale effort to classify MMR gene variants according to pathogenicity, based on variant and family characteristics on the one hand and results from various functional assays on the other hand [16]. A five-tiered classification of the International Agency for Research on Cancer was adopted since it is linked to clinical recommendations. Classes 5 and 4 indicate a “pathogenic” and “likely pathogenic” variant, respectively, implying that a causative mutation was detected that warrants surveillance according to full high-risk guidelines and qualifies for predictive testing of at-risk relatives. Nonsense and frameshift mutations constitute a majority (59 %) of class 5 and 4 variants [16]. Classes 2 and 1 indicate a “likely non-pathogenic” and “non-pathogenic” variant, respectively, suggesting that the test result was normal and is to be treated as “no mutation detected”. Intronic variants (42 %) as well as non-synonymous (29 %) and synonymous (18 %) missense variants are the main types of changes represented among class 2 and 1 variants [16]. Class 3 is synonymous with a variant of unknown significance (VUS) that requires a multilevel functional assessment for a reliable assignment of pathogenic significance and clinical treatment is case by case. Non-synonymous missense changes are abundant (68 %) among class 3 variants [16]. Figure 3 shows the breakdown of the main pathogenicity classes across each MMR gene, based on the germline mutation data deposited in the InSiGHT mutation database [16]. Pathogenic mutations (classes 4 + 5) constitute a majority (except for *MSH6* with a dominant class 3) and normal variants (classes 1 + 2) constitute a minority of all database variants for each gene. The share of VUSes is 31 % for all deposited variants in *MLH1*, 28 % for *MSH2*, 47 % for *MSH6*, and 26 % for *PMS2.*

### Unique versus recurrent mutations

Most MMR gene mutations are inherited from either parent and de novo mutations are rare (2.3 % [18]). A majority of all MMR gene mutations are unique, i.e. specific to a single family. However, some prevalent recurrent mutations are known and based on haplotype analysis, may arise de novo or alternatively, represent founder mutations [19]. Certain regions of MMR genes may be mutation-prone due to specific sequence characteristics. For example, c.942+3A>T, a splicing mutation in intron 5 of *MSH2* and one of the most frequently recurring MMR gene mutations worldwide, is likely to arise as a consequence of misalignment while replicating 26 consecutive adenines, of which the mutation-associated adenine is the first [20]. The same A_26_ repeat is part of BAT26, a key marker in MSI detection [21]. The c.942+3A>T mutation arises de novo in some populations [20] and represents a founder mutation in other populations [22]. Founder mutations originate from a single ancestor and become enriched in isolated populations [23]. Based on the extent of haplotype conservation, the age of founder mutations in MMR genes ranges from a few hundred to more than a thousand years [19]. To date, over 50 proven founder mutations in MMR genes are known from all over the world and may account for over 50 % of all LS families in some populations [19].

### Rates of mutation detection in LS families

Germline mutations in MMR genes are detectable in up to 88 % of LS families fulfilling the Amsterdam criteria [24, 25] and showing MSI in tumors [26, 27]. Smaller or atypical families and families not pre-screened for MMR deficiency in tumor tissues may display mutation frequencies of 10–40 % depending on the criteria of ascertainment [26, 28, 29]. A genetic point mutation is the predominant type of germline mutation in MMR genes in most populations [26, 27, 30]. Analysis of LS cohorts from several different geographic locations yielded a frequency of 15 % (68 unrelated kindreds out of 439) for large genomic rearrangements; among 48 different rearrangements, 29 affected *MSH2*, 13 *MLH1*, 2 *MSH6* and 4 *PMS2* [31]. A few percent of Lynch-suspected families with MMR-deficient tumors and negative for point mutations and large rearrangements are due to constitutional epimutations in MMR genes (see below).

Over half of MMR-deficient tumors that are not explained by germline mutations or (acquired or constitutional) promoter methylation of MMR genes (“Lynch-like syndrome”) were recently shown to arise as a consequence of somatic mutations in MMR genes [32–34] occasionally combined with *POLE/POLD1* defects [35], i.e. are non-hereditary as a rule. MMR gene mutations are very rare in families with MMR-proficient tumors, even if they meet the Amsterdam criteria (Familial Colorectal Cancer Type X) [36]. The genetic basis of Familial Colorectal Cancer Type X families seems heterogeneous and predisposing genes and mutations remain unknown in a majority [37–39].

### Constitutional epimutations in LS predisposition

Constitutional epimutation refers to hypermethylation at the promoter of one allele of a given gene leading to silencing of expression from that allele in all main somatic tissues. Constitutional epimutation of *MLH1* occurs in 2–3 % of mutation-negative Lynch-suspected families with silenced MLH1 expression in tumors [40–42]. Since constitutional epimutations are reversible during meiosis [43], epimutations segregate in a non-Mendelian fashion and are seldom associated with strong family histories of cancer. Epimutations secondary to genetic mutations constitute an exception and may arise on ancestral founding haplotypes [44]. The prevalence of *MLH1* constitutional epimutations in colorectal cancers lacking MLH1 expression and showing *MLH1* methylation in tumor tissue was reported to be 0 % among unselected cases and 16 % among cases fulfilling the revised Bethesda criteria [21], suggesting that testing for *MLH1* epimutations should regularly be restricted to the latter group of patients [45].

Constitutional epimutations of *MSH2* are secondary to deletions of the 3′ end of the *EPCAM* gene which make transcription of *EPCAM* read into the adjacent, structurally normal *MSH2* gene inducing its promoter to be methylated [46]. *EPCAM* deletion-associated *MSH2* epimutations vary a lot in frequency between populations depending on possible founder effects and may account for 10–40 % of families with absent MSH2 protein in tumors [42, 46]. Such epimutations show regular Mendelian transmission along with *EPCAM* deletion in pedigrees [46].

## Genotype–phenotype correlations

### Cancer risks associated with germline mutations in individual MMR genes

The lifetime risks of cancer are significantly higher in *MSH2* and *MLH1* mutation carriers compared to carriers of *MSH6* or *PMS2* mutations, which may reflect functional redundancy of MSH6 (with MSH3) and PMS2 (with MLH3 and PMS1) (see above). The lifetime risk by age 70 of any LS-associated cancer has been found to range between 57 % [47] and close to 80 % [48] for *MSH2* and 59 % [47] and ~65 % [48] for *MLH1*. For *MSH6*, lifetime risks of 25 % for males and females combined [47] and 24 % (males) and 40 % (females) [49] have been reported. Heterozygous *PMS2* mutation carriers may have a 25–32 % lifetime risk of any cancer [50].

Among the various cancers arising in *MSH2* and *MLH1* mutation carriers, the highest lifetime risk is for colorectal cancer, followed by endometrial cancer and other extracolonic cancers; moreover, *MSH2* mutations may be associated with higher risks of extracolonic cancers compared to *MLH1* mutations [48, 51]. Female carriers of *MSH6* mutations are at a higher risk of endometrial than colorectal cancer [47, 49, 52]. The same may be true for heterozygous carriers of *PMS2* mutations [50]. Furthermore, *MSH6* and *PMS2* mutations show reduced age-specific penetrance, resulting in higher average ages at onset of various cancers in *MSH6* [52] and *PMS2* [50, 53] carriers compared to *MSH2* or *MLH1* mutation carriers, although family- and/or mutation-specific variations exist. No clear-cut correlations have been observed between the type (e.g., truncating vs. missense) or location (e.g., relative to different functional domains) of a MMR gene mutation and clinical phenotype.

### LS tumor spectrum

A major puzzle in LS (in common with a majority of familial cancer syndromes) is the specific spectrum of tumors in constitutional mutation carriers. The Amsterdam II criteria [25] acknowledge cancers of the colon and rectum, endometrium, small bowel, ureter, and renal pelvis as LS-associated cancers, based on their significant overrepresentation in LS compared to the average population. Since these criteria were formulated, significantly increased standardized incidence ratios have repeatedly been reported for several other cancers as well, including cancers of the stomach, ovaries, and pancreas [54, 55]. Combined with molecular profiles characteristic of LS (e.g., consistent MMR protein loss or MSI [56–58], inclusion of these tumors in the LS spectrum seems justified. For breast cancer, the observed standardized incidence ratios vary from comparable to the average population [59, 60] to significantly elevated [54, 55], making it difficult to conclude whether or not breast cancer belongs to the LS spectrum. A recent comparative study on proven mutation carriers versus non-carriers did find a significant difference in the rate of MMR deficient breast carcinomas between those two groups (65 vs. 0 %, *P* < 0.001) [61]. Moreover, the age at onset in mutation carriers depended on the MMR status of their tumors (earlier onset if the tumor was MMR-deficient), suggesting a role for deficient MMR in breast cancer development in LS [61].

### Factors that may contribute to the LS tumor spectrum

**Fig. 4:**
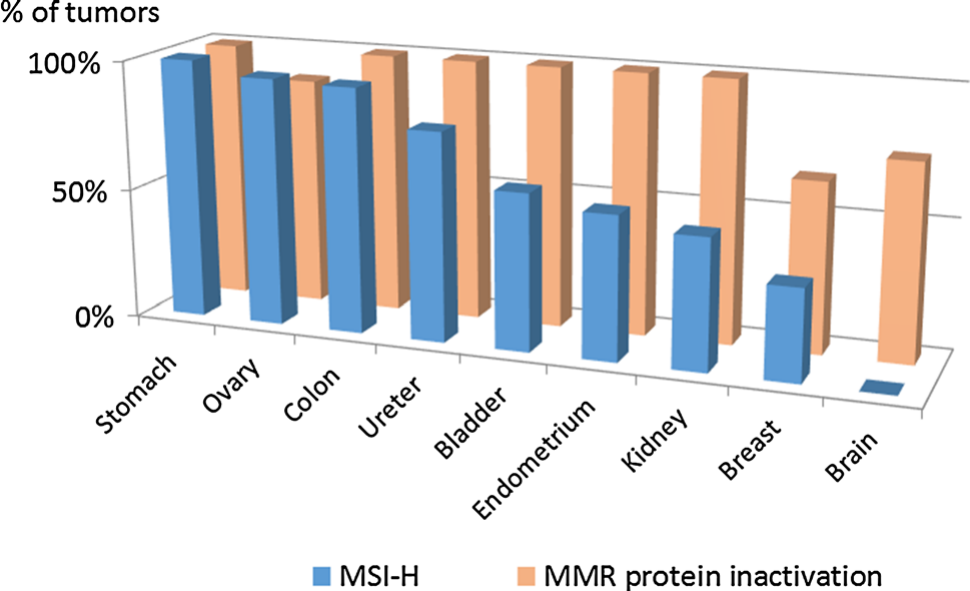
Tumor-specific patterns of MMR defects. Percentages of tumors with MSI-high and MMR protein inactivation among cancers arising in different organs in germline carriers of MMR gene mutations from a nation-wide registry [57, 61, 62] are shown

As described above, the individual MMR genes may be associated with somewhat different tumor spectra. In addition, a number of other factors may contribute to the LS tumor spectrum. Tissue-specific patterns of MMR deficiency in cancers from MMR gene mutation carriers (Fig. 4) may constitute one such factor. While immunohistochemical analysis of malignant tumors regularly demonstrates the absence of MMR protein corresponding to the gene mutant in the germline, the frequencies of tumors with MSI-high vary, being 80 % or above for stomach, ovary, colon, and ureter cancer, ~50 % for bladder, endometrium, and kidney cancer, and 35 % or below for breast and brain tumors [57, 61, 62]. Clonal heterogeneity is a feature of LS and sporadic MMR-deficient tumors [63, 64] and may in part explain the different frequencies of MSI between tumor types. Moreover, *BAT* markers show shorter allelic shifts in endometrial cancers compared to colorectal cancers from LS patients [65]. Such differences may be important considering the fact that genes with repetitive sequences in coding regions are mutation-prone in MMR-deficient cancers. Different genes confer selective advantage in different cancers, for example, the *TGFβ* superfamily is a mutational target in gastrointestinal cancers and *PTEN* in endometrial cancers [65, 66]. Tissue-specificity for MMR deficiency and genes targeted by failing MMR may therefore contribute to organ-selectivity.

Two lines of evidence imply that the dosage of the MMR gene or protein is important for phenotype. First, homozygosity or compound heterozygosity for germline mutation gives rise to a distinct syndrome, constitutional mismatch repair deficiency syndrome (CMMRD). Currently, 146 patients from 91 families with this syndrome are known [67]. Childhood cancers of the hematological system and brain, signs of neurofibromatosis type 1 (café-au-lait spots) and Turcot syndrome (coexistence of colorectal tumor and brain tumor) are common manifestations of CMMRD. The peculiar tumor spectrum may reflect the sensitivity of particular (e.g. neural and hematological) progenitor cells to MMR deficiency via specific somatic target genes (*NF1* mutation [68]). *PMS2* and *MSH6* predominate over *MSH2* and *MLH1* as genes underlying CMMRD [67]. Contrary to traditional LS with heterozygous MMR gene mutations, CMMRD patients lack expression of the MMR protein(s) in question not only in cancer tissue but in normal tissue as well. MSI in tumor tissues varies in the same way as in conventional LS (present in gastrointestinal tumors but absent in brain tumors as shown for LS in Fig. 4). Standard techniques cannot usually detect MSI in peripheral blood lymphocytes because of clonal heterogeneity, whereas immortalized lymphoblastoid cells may reveal a MSI phenotype [67].

Another line of evidence in support of the importance of MMR gene or protein dosage comes from observations that the presence of the wild-type copy of a MMR gene in somatic cells is not always sufficient for a normal function (haploinsufficiency). While MMR genes usually comply with Knudson’s two-hit hypothesis for tumor suppressor genes [69] as evidenced by the lack of the responsible MMR protein in LS-associated cancers (biallelic inactivation), colorectal adenoma development seems possible in LS even if the wild-type allele of the predisposing MMR gene is retained [70, 71]. Other possible molecular “hits”, such as epigenetic inactivation of tumor suppressor genes [71] may contribute to tumor initiation in such haploinsufficient cells. Haploinsufficiency may be function-specific; for example, it has been demonstrated that DNA damage signaling requires a higher dosage of MMR protein than the repair function [72]. Failure of apoptosis signaling likely provides MMR-deficient cells with selective advantage needed for tumorigenesis [73]. Different organs may have different requirements for MMR gene dosage [61, 74], which may influence their susceptibility to tumor development.

As discussed above, the MMR system recognizes several other types of DNA damage besides replication errors, including oxidative [3] and alkylating [4] damage, as well as heterocyclic amine (e.g., PhIP) DNA adducts [75]. Such damage can be exogenous (e.g., PhIP is a cooked meat-derived mutagen [75]) or endogenous (e.g., oxidation resulting from normal cellular metabolism or inflammation [76]). Organs commonly exposed to such damage, such as the gastrointestinal tract and endometrial epithelium, would obviously be at elevated risk of cancer development, especially in individuals with deficient MMR. Unhealthy diet (“snack” pattern [77] and tobacco smoking [78] have been shown to increase colorectal adenoma risk in MMR gene mutation carriers, which might imply a reduced capacity to correct dietary and tobacco-associated damage.

Tumor development initiated by replication errors or carcinogen-induced mutations may proceed at different rates in different tissues depending on their proliferative activity [79]. Colon and many other epithelial cells have fast turnovers, and there may be less time to repair replication errors if cell cycles are short [80]. Furthermore, in colon and other epithelial organs, stem cell divisions continue throughout life. Hematopoietic tissue, too, is highly proliferative, but may be less prone to malignancies because of fewer stem cell divisions during lifetime [80]. These general concepts are in agreement with the fact that a majority of LS-associated tumors are epithelial and that MMR deficiency also predisposes to hematological malignancies, but mainly in the context of CMMRD only.

Frameshift mutations typical of MMR deficiency result in the formation of neoantigens recognized by the immune system. Consequently, colon, gynecological, and other tumors from LS patients display high levels of tumor infiltrating lymphocytes (TILs) [81, 82]. The abundance of CD8+ cells (dominant in TILs) is a good prognostic sign in LS and sporadic cancers [81, 83]. On the other hand, frameshift mutations may also affect cell surface proteins responsible for antigen processing and presentation and thereby facilitate escape from immune surveillance [84]. Varying frequencies of MMR defects in different types of tumors (Fig. 4), combined with possible variations in the inherent efficacy of immune surveillance in different organs, may thus contribute to organ-specific tumor susceptibility in LS.

## Concluding remarks

Research on LS conducted to date has greatly advanced our understanding of the significance of the MMR system in human cancer. Yet, many essential questions wait for definitive answers regarding the mechanisms of tumorigenesis (e.g., two-hit inactivation vs. haploinsufficiency) and the complex relationship between genotype and phenotype (e.g., genetic vs. non-genetic influences; unequivocal definition of the LS tumor spectrum) to mention a few. Targeted gene panels based on next-generation sequencing [85, 86] will be changing the approach to screen for predisposing mutations in LS and other hereditary disorders in the coming years. The simultaneous screening of all LS-associated MMR genes and other possible susceptibility genes in LS-suspected cases is likely to more accurately define the spectrum of genes and mutations predisposing to LS and the population incidence of LS. Next-generation sequencing of the whole exomes and genomes is also anticipated to provide new insights into the genetic basis of colon cancer families that are unrelated to MMR defects (Familial Colorectal Cancer Type X) [38, 87, 88].

Comprehensive genetic, epigenetic, and expressional cataloguing of tumor alterations in analogy to ongoing efforts on sporadic cancers (e.g., by the Cancer Genome Atlas Network [89, 90]) will be useful to define the developmental mechanisms of colonic and extracolonic tumors in LS and to better understand the molecular basis of organ-specific tumor susceptibility. Targeted studies [57, 91] have already revealed distinct mutational patterns in LS tumors that may explain the disease outcome and be clinically actionable. When linked to clinical parameters, comprehensive molecular profiles of constitutional and tumor tissues will be informative to establish clinical correlations of molecular aberrations and facilitate the management of individuals with LS.
